# Hazard and risk assessment strategies for nanoparticle exposures: how far have we come in the past 10 years?

**DOI:** 10.12688/f1000research.12691.1

**Published:** 2018-03-26

**Authors:** David B Warheit

**Affiliations:** 1 The Chemours Company, Wilmington, Delaware, USA

**Keywords:** Nanotechnology, nanosafety, nanoparticle exposures

## Abstract

Nanotechnology is an emerging, cross-disciplinary technology designed to create and synthesize new materials at the nanoscale (generally defined as a particle size range of ≤10 ^-9^ meters) to generate innovative or altered material properties. The particle properties can be modified to promote different and more flexible applications, resulting in consumer benefits, particularly in medical, cosmetic, and industrial applications. As this applied science matures and flourishes, concerns have arisen regarding potential health effects of exposures to untested materials, as many newly developed products have not been adequately evaluated. Indeed, it is necessary to ensure that societal and commercial advantages are not outweighed by potential human health or environmental disadvantages. Therefore, a variety of international planning activities or research efforts have been proposed or implemented, particularly in the European Union and United States, with the expectation that significant advances will be made in understanding potential hazards related to exposures in the occupational and/or consumer environments. One of the first conclusions reached regarding hazardous effects of nanoparticles stemmed from the findings of early pulmonary toxicology studies, suggesting that lung exposures to ultrafine particles were more toxic than those to larger, fine-sized particles of similar chemistry. This review documents some of the conceptual planning efforts, implementation strategies/activities, and research accomplishments over the past 10 years or so. It also highlights (in this author’s opinion) some shortcomings in the research efforts and accomplishments over the same duration.

In general, much progress has been made in developing and implementing environmental, health, and safety research-based protocols for addressing nanosafety issues. However, challenges remain in adequately investigating health effects given 1) many different nanomaterial types, 2) various potential routes of exposure, 3) nanomaterial characterization issues, 4) limitations in research methodologies, such as time-course and dose-response issues, and 5) inadequate *in vitro* methodologies for *in vivo *standardized, guideline toxicity testing.

## Introduction

Nanotechnology continues to be an emerging multidisciplinary science platform that is often defined as creating products and applications based primarily upon the synthesis of molecules in the nanoscale (10 ^-9^ meter) size range. The term “nano” arises from the Greek term, referring to “dwarf”. What makes the technology intriguing is that particle properties may often become altered as they are reduced below 100 nm in size. For example, gold particles may transform to blue or red pigments. Electrical insulating functions of certain particle types may become conductors at the nanoscale. In short, particle properties can be modified to promote different and more flexible applications, resulting in consumer benefits, particularly in the areas of medical and industrial applications. From a material science perspective, this represents an exciting challenge because as the particulate material size decreases in the direction of the nanoscale (particularly below 100 nm), the physical properties of particles are known to be altered and can be harnessed to create new products and applications ^1^.

Because of the economic potential, commercializing various types of engineered nanomaterials for a range of applications, including industrial, consumer, medical, and diagnostic, clearly presents a challenge for companies and regulators to ensure the development of safe and effective products for consumers. Therefore, assessments of potential hazards associated with this technology and corresponding products have become emerging areas for health risk assessments. This understanding represents an element of the broad engagement process with stakeholders and potential customers with regard to environmental health and safety issues.

There exists an interesting conundrum with respect to natural versus “synthetic” nanotechnology. From the outset, the existence of “naturally” derived nanoparticle types has long been recognized, including particulate components of combustion, concomitant with commercial products that were previously unrecognized by the public as being in the nanoscale range, e.g. carbon black particles (natural nanoscale carbon particulates), nanoscale titanium dioxide particulates, or zinc oxide particles for health-related sunscreen applications. However, as the commercialization and promotion of the technology has become more widespread to consumers, concerns have been raised about safety issues with questions relating to health effects and impacts on the environment. Questions initially were raised about the inhalation route of exposure, i.e. can inhaled ultrafine particles have adverse pulmonary impacts when compared to fine particles? But, equally important, the safety of the other two major routes of exposure, i.e. ingestion/oral and dermal, has also been raised. Therefore, it is critical to assess the health impacts of nanoparticle exposures for all routes when considering exposures to both occupational workers and consumers.

During the past 10 years, several research strategies and challenges have been proposed to facilitate and direct “verification”, i.e. the safe handling of and exposures to individual forms of nanoparticles and nanotechnology in general. A major problem is that all of the different nanoparticle types cannot be effectively evaluated for safety and environmental effects in a timely manner owing to 1) the vast numbers of different nanoparticle types, 2) the numerous variations within specific nanoparticle types (e.g. there are many different nanoscale forms of carbon: carbon black particles, fullerenes, single-walled carbon nanotubes, multi-walled carbon nanotubes [MWCNTs], carbon nanofibers [CNFs], etc.), 3) the overwhelming expense required to adequately test each individual nanoparticle type, 4) the inadequate time that would be required to test each of the nanoparticle types (e.g. acute tests may be studied for a short period of time, perhaps 24 hours, but these types of studies do not represent longer-term exposures to humans or in the environment, i.e. subchronic or chronic lifetime studies, which in rats can be 2 years, and 5) the vast expense can easily cost approximately 2 million dollars for a single chronic inhalation study.

As a result of this large but important dilemma, efforts have been expended by numerous authors, groups of research investigators, entire workshops, and millions of dollars/euros of grant funds to provide “guidance” on developing research health and safety challenges and strategies and to support or sponsor inter-laboratory research validation efforts as well as funding for investigators to study the hazards and environmental impacts (toxicology) of different nanoparticle exposures. It is hoped that an understanding of both health effects and environmental hazards of nanoparticle exposures (pulmonary, oral, or dermal) as well as environmental effects could lead to improved health and environmental risk evaluations.

### Why is this brief retrospective important to the reader?

Given the potential beneficial impact of nanotechnological innovations in our society, this brief review identifies and documents many of the initial conceptual grand challenges, discussions, workshop activities, and, finally, collective research efforts that have been expended, organized, promulgated, and published in an attempt to encourage, rather than to discourage, the development of the technology; and to foster the implementation of risk assessment strategies following nanoparticle exposures relative to the normal routes of entry, i.e. inhalation, oral, and dermal. Basically, the question is how best to approach this difficult but important set of issues.

Some of the original research efforts, discussions, and challenges/workshop issues/conclusions that have been proposed and implemented during the past 10 years or so are presented; for example, some of the more prominent European Commission FP7-sponsored programs on nanosafety issues followed by some pertinent conclusions of the US National Academy of Science Committee on Research Progress of Environmental Health and Safety Aspects of Engineered Nanomaterials. In addition, the implementation components of a risk framework for nanomaterials is briefly outlined. The NanoRisk framework was a collective 2-year effort fostered by ongoing discussions of a non-governmental organization (NGO) (Environmental Defense Fund) and a company synthesizing and commercializing products derived at the nanoscale (the DuPont company). The objectives of the framework were to develop and produce a systematic and disciplined procedure to identify, manage, and reduce the possible environmental safety and health risks associated with engineered nanomaterials throughout every stage of a product’s life-cycle. The scope of the framework provides an informed counsel regarding many of the essential issues that should be considered by an organization when developing the applications of nanomaterials and regarding research findings in order to deliver solid risk evaluations and risk-management decisions. The target audiences represent companies and research institutions that are actively working with nanomaterials to develop applications or products.

For completeness, additional sections of this mini-review include discussion of important and controversial issues related to 1) the evaluation of the strengths and weaknesses of the entire scientific literature on nanosafety research over the past 10 years by an expert who has read and critically assessed over 6,000 studies out of more than 10,000 publications selected on human health effects or biological studies; and 2) important discussions on “biokinetics and biodistribution” of nanomaterials following pulmonary, dermal, or oral exposures (i.e. where do the particles travel within the body following initial exposures?).

Finally, a study design and the implementation of a subchronic (13-week) inhalation toxicity study with CNFs in rats are described. This discussion serves to demonstrate and emphasize the difficult challenges, time dependency, expenses, and toxicological complexity that are required to generate meaningful data and provide accurate perspectives on the issues related to developing adequate risk assessments for the variety of nanomaterial types that currently exist in commerce and to which workers and consumers are exposed.

## Initial grand challenges posed (2006) and revisiting progress 10 years later (2016)

One of the earlier efforts to stimulate risk-related research was proposed as a series of “Grand Challenges” by Maynard and colleagues in 2006 ^2^. These investigators recommended five generalized grand challenges for nanotechnology research, which were conceptually designed to serve as a blueprint to stimulate nanosafety research strategies.

The five grand challenges were described in the following manner and they addressed the following needs:

1) Advancement of equipment over the next 5–15 years to estimate or determine aerosolized and water exposures to engineered nanomaterials
2) Techniques and approaches to assess the hazards of engineered nanomaterials
3) Developing predictive models to gauge the potential hazardous effects of nanomaterial exposures on environmental and human health systems (environmental, health, and safety [EHS])
4) Developing methods for estimating the EHS impacts of nanomaterial exposures over a lifespan
5) Facilitating methodologies to assess strategically programs that could be utilized to implement relevant risk-focused research

Following the publication of five grand challenges for developing “safe nanotechnology” in 2006, two of the authors (Maynard and Aitken) ^3^ assessed the progress made during the subsequent 10-year period. It was concluded that appreciable progress had been made in the advancement and funding of important programs to initiate health-related research regarding the safety of nanomaterials. Specific organizations that were identified included 1) the US-based National Academy of Sciences, which published a comprehensive research plan for nanosafety research, 2) the European Union, as shown by FP7 and Horizon 2020 programs, which sponsored numerous research-based consortia, and 3) the Organization for Economic Cooperation and Development (OECD) projects, which developed protocols to standardize and validate test methods for conducting toxicity studies on nanomaterials. It should be noted that the success of this first initiative was related primarily to the funding of safety-related projects and could be measured, in part, by the numbers of publications addressing two of the previously listed grand challenges: 1) hazard studies on nanomaterials and 2) life-cycle assessments. In contrast, however, little significant progress was made on developing instruments to monitor airborne or waterborne engineered nanomaterials or predictive methodologies for modeling applications.

## ECETOC workshop: testing strategies to establish the safety of nanomaterials

During the same time, the European Centre for Ecotoxicology and Toxicology of Chemicals (ECETOC) convened a workshop in Spain of 70 scientific and clinical experts from all relevant sectors (academia, research institutes, governmental agencies, industry, and NGOs) in November 2005 ^4^. The participants were charged with addressing the following primary questions related to nanomaterial health effects: what can we do today and what do we need tomorrow? The three major topics to be addressed at the workshop were 1) the need for enhanced efforts in the characterization of nanoparticles, 2) development of methods for the evaluation of aerosolized and internal exposures to nanomaterials, and 3) evaluation of the hazard potential following pulmonary or dermal routes of exposures. The workshop participants concluded that physical factors can influence toxicity, including particle composition, surface area, and characteristics such as size and shape. With regard to a testing approach for human health effects, a first step might be to gauge potency hazards, and it was recommended to include *in vitro* screening assessment strategies to evaluate possible reactivity, biomarkers, inflammation, or cellular uptake indices.

## European FP7 projects and publications

### ITS NANO: prioritizing nanosafety research to develop a stakeholder-driven intelligent testing strategy

A series of workshops were convened for expert stakeholder groups (i.e. government, industry, academia, funders, and NGOs) with the intention of proposing, discussing, and ultimately conceptually implementing an “intelligent testing strategy” (ITS) designed to assess the risk of nanomaterials on a case-by-case basis ^5^. The ITS framework was considered to be a process that promotes the risk of nanomaterials to be evaluated accurately, effectively, and efficiently, thereby obviating the need to test each and every nanomaterial type, on a case-by-case basis. The major topics were determined, by consensus, to represent 10 different commercially available nanomaterials. Physicochemical characterization, exposure identification, hazard assessments, and modeling—deemed to represent key priorities for research areas—were modeled in a “stepping stone” matrix. This stepping stone matrix was represented by hexagonal diagrams which served to provide tools for individual stakeholders to formulate enhanced reliability criteria for developing *in silico* approaches. A manuscript written to summarize the meeting results described an appraisal of how this particular framework compares with current risk assessment approaches and how future methodologies could adapt to accommodate new approaches. According to the authors, the ITS-NANO project expressed a detailed, flexible, and stakeholder-driven research strategy tool which describes and prioritizes specific research needs for dealing with a wide variety of nanomaterial types, including newly discovered or synthesized materials.

### Engineered nanomaterial risk: lesson learnt from completed nanotoxicology studies—potential solutions and future challenges

This review paper summarized the results from the European project entitled Particle Risk, which was one of the first multidisciplinary projects funded by the European Commission’s Framework Programme (dealing with implications of nanomaterial exposures on human health) ^6^. The authors evaluated the findings of numerous nanotoxicology-based research publications up to that date and reflected on the progress and advancement made regarding risk assessment conclusions for nanomaterial exposures. Several subtopics were described and evaluated, including 1) nanomaterial selection, 2) nanomaterial physicochemical characterization, 3) nanomaterial dispersion techniques, 4) dose level selection and concentrations for implementing experiments, 5) identification of target organ sites and endpoints, 6) development of animal alternative methodologies, and 7) nanomaterial risk assessment methodologies. The authors considered the value of this review article to be in its informing of a discussion of these various important issues and providing guidance on improving upon the utility of current methods, thus achieving and fulfilling enhanced relevant strategies and experimental designs which could better inform upon the wide range of relevant nanomaterial risk assessments.

### A multi-laboratory toxicological assessment of a panel of 10 engineered nanomaterials to human health—ENPRA project: the highlights, limitations, and current and future challenges

ENPRA (Risk Assessment of Engineered NanoParticles) was an early multidisciplinary European Commission FP7-funded project designed to assess the risks to human health following nanomaterial exposure specifically when considering pulmonary, cardiovascular, hepatic, renal, and developmental systems ^7^. The project reviewed and considered results and interpretations of a wide range of *in vitro* and *in vivo* studies. The main objectives of the ENPRA project were 1) to assess the physicochemical characteristics of 10 commercially available nanomaterials, 2) to evaluate the hazards of these nanomaterials using *in vitro* toxicology testing methods with cells sourced from pulmonary, cardiovascular, hepatic, renal, and developmental systems and to examine five potential mechanisms of toxicity including cytotoxicity, oxidative stress, inflammation and immune response, genotoxicity, and potential fibrogenicity, 3) to confirm *in vitro* findings with relevant *in vivo* testing using acute (24-hour) intratracheal exposure of mice, 4) to utilize data generated from this project in an “exposure–dose-response” relationship paradigm via “mathematical modeling”, and 5) to develop and implement a strategy for the dissemination of findings for risk assessment and risk management determinations.

The project generated substantial fruitful data and, in some cases, achieved similar comparative findings utilizing both *in vitro* and *in vivo* methodologies. One of the major goals was to apply the ENPRA completed objectives to achieve a framework for quantitative risk assessment, particularly in occupational workplaces. Alternatively, some of the shortcomings of the ENPRA projects were related to the use of non-physiologically relevant intratracheal particle exposures (versus more physiologically relevant inhalation exposure designs) concomitant with the lack of a full time-course experimental regimen (i.e. the protocol for the studies utilized only a 24-hour post-exposure regimen) which did not permit a full accounting of risk assessment evaluations.

### Comprehensive *in vitro* toxicity testing of a panel of representative oxide nanomaterials: first steps towards an intelligent testing strategy

The FP7-MARINA (Managing Risks of Nanomaterials) project aimed to identify and evaluate *in vitro* test methods for hazard of toxicity evaluation in order to promote the development of an ITS (see above description) ^8^. In protocols similar to other European Commission FP7 projects (using round-robin laboratory comparisons for uniformity of results), six representative oxide-type nanomaterials provided by the European Commission Joint Research Center (JRC) were tested using *in vitro*-type methodologies in nine different research laboratories using 12 different cellular models incorporating six different target organ systems. According to the authors, a hazard ranking could be established for the representative nanomaterials tested (a form of zinc oxide [ZnO] NM-110, ZnO NM-111 > silicon dioxide [SiO _2_] NM-203 > SiO _2_ NM-200 > TiO _2_ NM-104 >TiO _2_ NM-103). The investigators proposed that their testing approach could be implemented for the development of an ITS strategy suitable for nanomaterial risk assessments.

## Consensus opinions on *in vitro* approaches to assessing pulmonary fibrogenic potential of aerosolized nanomaterials

More recent attempts have been made to obtain consensus on *in vitro* approaches to evaluate the pulmonary fibrogenic potential of aerosolized nanomaterials ^9^. In an attempt to develop substitute *in vitro* strategies to replace 90-day inhalation studies, given the monetary, ethical, and scientific concerns with this *in vivo* test, an international expert panel was convened in Washington, DC, to discuss and design possible alternative approaches to assess the inhalation toxicity of MWCNTs using alternative, *in vitro*-type strategies. Inhalation exposures to MWCNTs in rats are known to produce pulmonary fibrosis as a key adverse outcome. Given that the development of lung fibrotic responses can require weeks or months to develop *in vivo*, it was postulated that an *in vitro* test system might serve to replace inhalation studies in a more rapid fashion by measuring and documenting pro-fibrotic precursors/mediators such as relevant cytokines and growth factors that have been implicated in the development of adverse lung outcomes. Accordingly, the workshop discussions dealt primarily with recommendations for designing and monitoring necessary indicators for such an *in vitro* system, utilizing relevant co-cultured lung cells, preferably exposed at a relevant *in vitro* aerosolized exposure system and utilizing air–liquid interface (ALI) methodologies. The investigators acknowledged that future planning is a necessary prerequisite for ultimate substitution of inhalation studies in rats with *in vitro* methodologies; the current effort represents only a very early step in the process and has many hurdles ahead for any serious consideration of *in vitro* substitution as a viable and achievable system for replacing inhalation toxicity studies.

## National Academy of Sciences committee opinions and recommendations on research progress on EHS aspects of engineered nanomaterials

Recently, a National Academy of Sciences committee undertook an assessment of research progress on EHS aspects of engineered nanomaterials ^10^. A major focus of the committee’s report was to provide consideration and guidance on the development of research strategies for developing basic toxicity studies for the wide variety of current and future nanomaterials, owing, in large part, to time and expense considerations. Obviously, there exists a critical need for implementing reliable and validated screening tools to identify and confirm toxicity pathways for health and environmental effects and to address important mechanistic issues, given that the reliable testing of individual nanoparticle types is not practical. In addition, toxicity results obtained from a variety of *in vitro* studies often have limited value for determining relevant health hazards, in large part because toxicological studies with engineered nanomaterials are often conducted at extremely higher doses than might be encountered in real-world exposures, concomitant with a focus on short-term toxicological responses. Accordingly, time-course experiments would better reflect potential realistic effects following exposures to nanomaterial types.

Alternatively, given the limitations of animal testing going forward, and with a greater resistance to conducting *in vivo* experiments, the application of a spectrum of *in vitro* investigations could provide some useful mechanistic insights into toxicity pathways, although several shortcomings to current *in vitro* methodologies need to be addressed. Optimizing the relevance of *in vitro* studies to real-world toxicity considerations should require the utilization of experimental designs that require dose-response behaviors over a full range of doses and should also require the duration of exposure using relevant cell types (focusing on route of exposure regimens), including time-course evaluations concomitant with comparisons and validation with corresponding *in vivo* systems.

To summarize the suggested framework and research strategy, implementation of the following considerations would significantly improve the methodology for the development of reliable and validated screening tools:

1) Rigorous physicochemical characterization of nanoparticle types and behavior through the life-cycle
2) Dose-response characterization and careful attention to reliable dosimetry at relevant human exposure levels
3) Particularly for *in vitro* studies, selection of relevant cell types and cell models that reflect the route of human exposures
4) Time-course assessments that span acute to chronic exposure durations
5) Application of proper benchmark controls to improve the interpretation of toxicological outcomes ^10, 11^

## Additional *in vitro* approaches

The lung is a complicated organ system made up of a number of different cell types (including type I and II alveolar epithelial cells, macrophages, interstitial cells, and vascular cells); thus, the pulmonary microenvironment and the complex interactions in the lung which occur following inhalation and subsequent deposition of particles are difficult to simulate when employing *in vitro* techniques. In addition, numerous studies have reported *in vitro* toxicity results using only single cell types (e.g. A549 lung epithelial cells or lung or peritoneal macrophages) ^10^. More recently, complex *in vitro* systems are being developed, which can, in part, better simulate particle phagocytosis, an important aspect in the simulation of lung defense responses to particles ^11– 13^. The development of such *in vitro* techniques expedites the transition from the current animal-based inhalation testing system to one that is based primarily on human cell lines and *in vitro* assays. Such reproducible, accurate, and validated cell-based *in vitro* screening assays for assessing pulmonary and genetic toxicity will have important benefits (i.e. screening more compounds in a faster, more reliable, and less expensive manner) and may provide experimental designs to address mechanistic questions.

To improve the efficacy of an *in vitro* methodological approach, several steps have been designed to better represent the physiology of the distal lung microenvironment, concomitant with the implementation of an aerosol exposure system. Accordingly, this transition from a traditional “submerged” cell culture system to a more physiologically relevant ALI cell culture system, using Transwell® Permeable Support devices (microporous membranes), is useful for providing and maintaining pulmonary cell co-cultures (of rat lung epithelial cells and alveolar macrophages) and exposing the co-cultures to particulate aerosols in a humidified atmosphere.

Many fundamental issues require consideration for optimization when transitioning from an animal inhalation toxicity set-up to an *in vitro* aerosol pulmonary toxicity study. These considerations include, but are not limited to, the following actions:

- Determining the cell types to be used in a co-culture or tri-culture system in order to better simulate the lung microenvironment
- Transitioning from primary cell types collected from animals to immortalized cells derived from cell lines and tested for biological functionality
- Determining the number or ratio of lung epithelial cells to alveolar macrophages in a co-culture plate
- Determining the aerosol generation method for particles/nanoparticles and appropriate, reproducible, and quantifiable dose metrics to be utilized for particle inhalation deposition assessments and for comparisons of the results of one study to another

## The NanoCare project

The NanoCare project (2006 to 2009) was one of the first big projects (before the European Commission started with the increase of the funding money within the seventh framework program) to demonstrate the good cooperation between industry and academia and resulted in some very important results concerning the fiber paradigm and metal oxides (the complete list of publications can be found here: https://www.nanopartikel.info/en/projects/completed-projects/nanocare/ver-nanocare).

As a result of this activity, a funding program called the same (NanoCare) was born and is still running in Germany with many projects funded (listed here: https://www.nanopartikel.info/en/projects). All information is combined within a specific website ( www.nanoobjects.info) which delivers not only a literature criteria checklist but also a summary of SOPs to be used by everyone.

## Evaluating the risks associated with nanomaterial exposures: the NanoRisk framework

A NanoRisk framework was conceptualized and promoted by two organizations, which would appear to have adversarial interests, specifically a NGO (Environmental Defense Fund) and a commercial company (the DuPont company), which commercializes products containing nanomaterials ^14^. The NanoRisk framework was a collective effort designed to pre-emptively formulate a systematic process to investigate EHS risks associated with exposures to products containing engineered nanoparticles. An early assessment of EHS hazards and risks often occurs in the absence of exposure assessments in the occupational workplace or in the environment. Therefore, exposure evaluations are frequently estimated, predicated upon informed estimation of the product’s life-cycle. To gain the necessary information, the framework should be composed in part of a base set of mammalian and environmentally based toxicity studies in order to achieve a reasonable evaluation of the mammalian and environmental hazards.

The NanoRisk framework is composed of six fundamental measures which align with stages of development and is a repetitive process. This framework can be downloaded at the following website address: www.nanoriskframework.com. Briefly, the six steps are outlined below:

Step 1. Careful physicochemical characterization of the material
Step 2. Delineate life-cycle(s)
  2A. Nanomaterial physicochemical properties
  2B. Nanoparticle toxicity findings
  2C. Nanoparticle exposures
Step 3. Assess risks
Step 4. Evaluation of risk management
Step 5. Decide, document, and act
Step 6. Review findings and readjust

**Figure 1.**
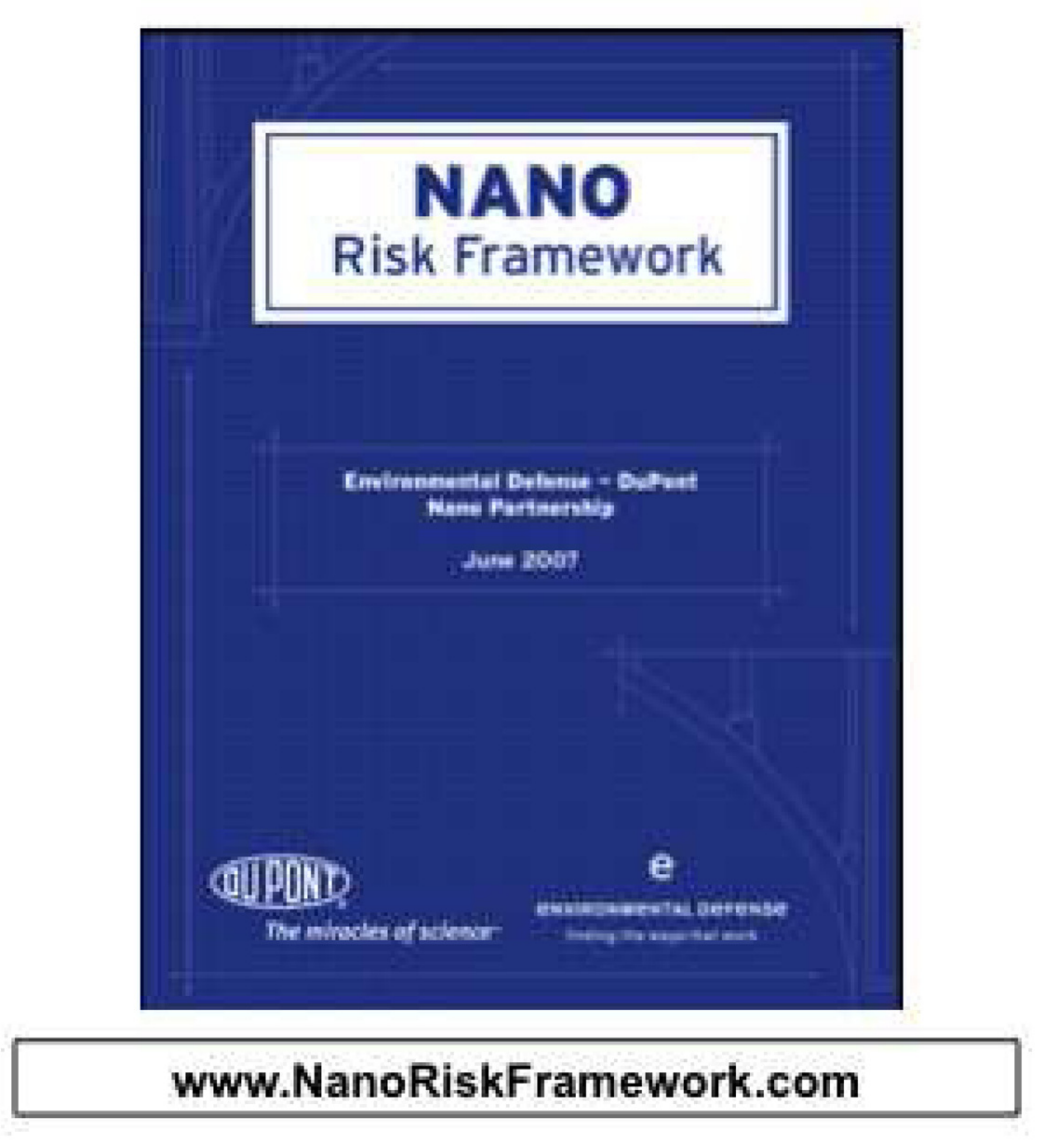
The Nano Risk Framework document.

**Figure 2.**
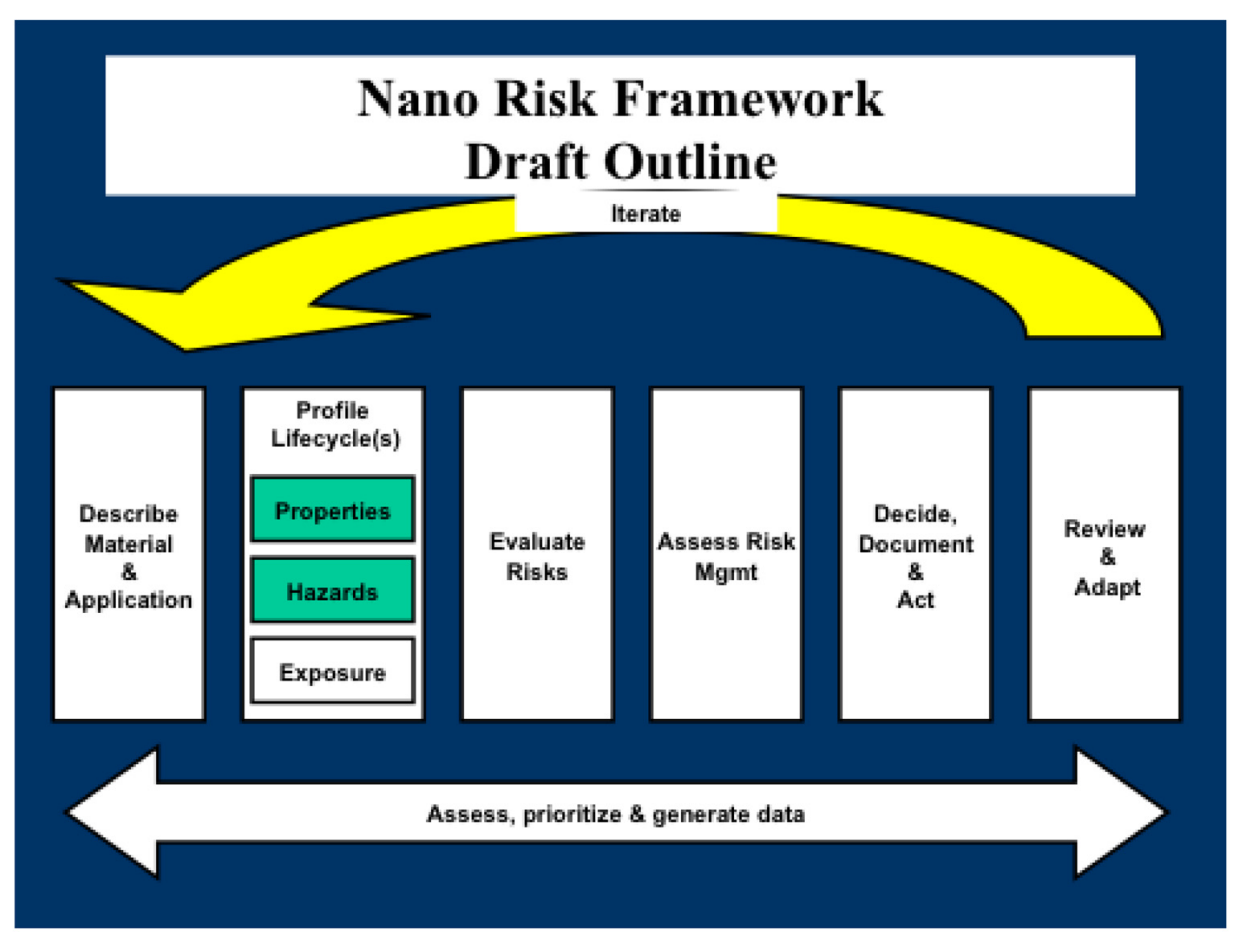
NanoRisk framework draft outline. The outline of iterative steps in the NanoRisk framework, commencing with a description of the material (i.e. robust physicochemical characterization) and application, continuing to the Review and Adapt step.

Figure 1 depicts the cover of this document, which can be downloaded at the website www.NanoRiskFramework.com. Figure 2 illustrates the iterative steps in the framework with the emphasis on profiling the life-cycle and focusing on the material properties and hazard effects.

**Figure 3.**
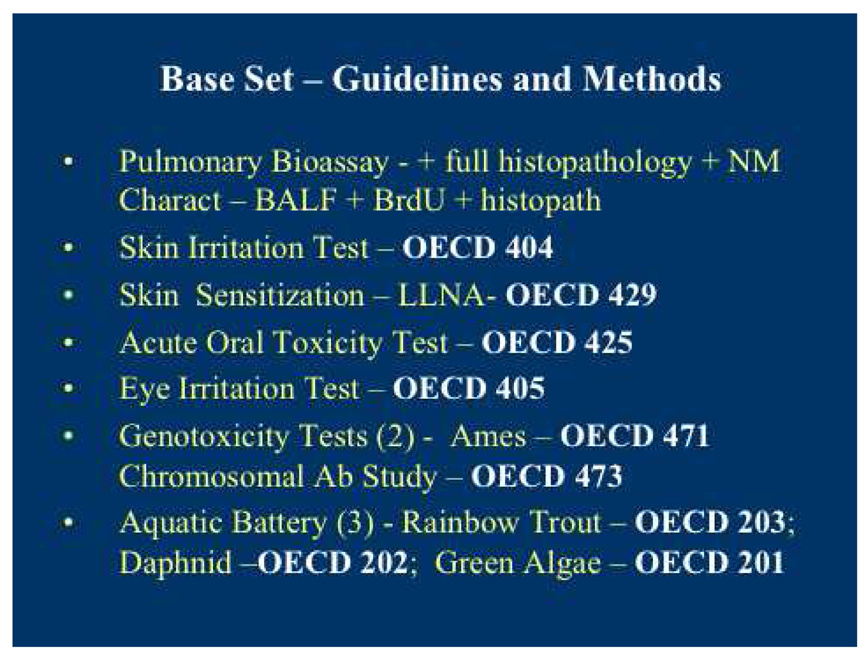
The guideline and methods included with the base set of toxicity studies. The base set describes mammalian toxicity studies, including pulmonary bioassay studies, skin irritation and sensitization tests, acute oral toxicity and eye irritation, and genotoxicity studies, along with ecotoxicological/aquatic battery of studies, including vertebrate, invertebrate, and green algae studies. Ab, aquatic battery; BALF, bronchoalveolar lavage fluid; BrdU, bromodeoxyuridine; LLNA, local lymph node assay.

**Figure 4.**
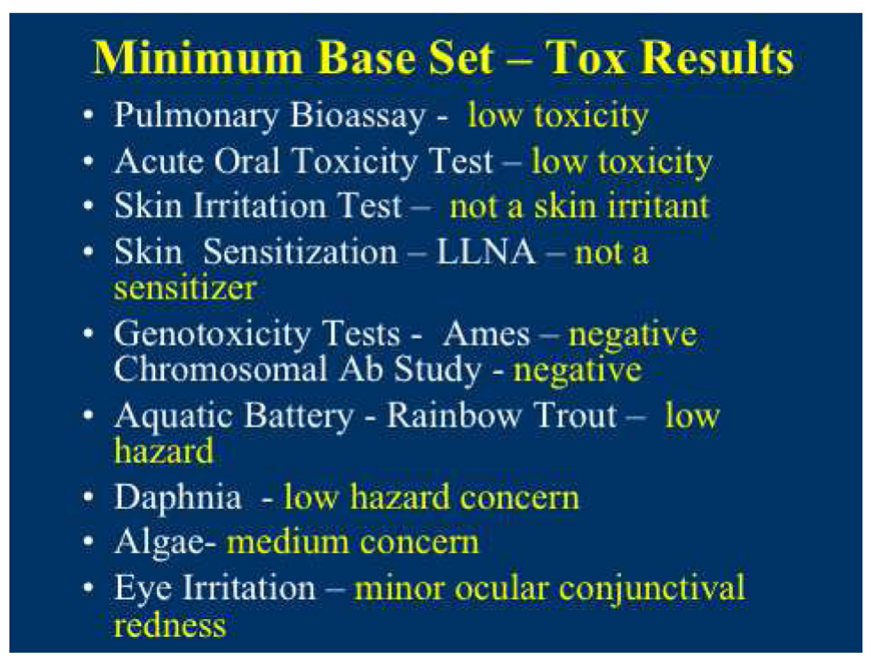
Minimum base set – tox results. The hazard results from the minimum base set studies on ultrafine TiO _2_ particles. Most of the mammalian or ecotoxicology results demonstrated no or low hazards following exposures to ultrafine TiO _2_ particles.

With respect to the hazard component of the framework with a new material, the hazard findings of a base set of toxicity tests on a newly developed, well-characterized, ultrafine rutile-type TiO _2_ (uf-TiO _2_) particle type were reported by Warheit *et al.* ^15^. These hazard assessments were composed by focusing upon general potential routes, including acute lung instillation studies, oral toxicity tests, dermal irritation and sensitization investigations, acute ocular (eye) irritation tests, genetic toxicology assessments, and screening aquatic toxicity guideline studies. Lung bioassay tests were conducted using a well-designed protocol, including dose-response and time-course parameters. The acute dermal irritation tests were carried out using rabbits according to standardized OECD 404 guidelines. The local lymph node assay in mice was performed to investigate skin sensitization (OECD 429 guideline). The acute oral toxicity test was conducted in rats according to a standardized OECD 425 guideline. An *in vitro* Ames mutagenicity assay was conducted using a standardized bacterial reverse mutation test (OECD 471) concomitant with an *in vitro* mammalian chromosome aberration test using Chinese hamster ovary cells (OECD 473). Acute aquatic toxicity assays were carried out to test for potential environmental effects using prototypical aquatic organisms, including rainbow trout (fish), daphnia (invertebrates), and green algae. Figure 3 and Figure 4 list the base set test guideline and methods as well as the OECD test references. ( Figure 4 lists the toxicity results of each of the health effects and environmental effects findings. To summarize, based upon a compilation of mammalian and aquatic toxicity evaluations, the summary outcome revealed a low hazard potential in both tested mammals concomitant with aquatic (environmental) species following short-term exposures ^15^ ( Figure 4). A considered review of all of the data strongly indicates that the commercialization and potential exposure of nanomaterials in this product would not result in adverse health effects.

## Subchronic inhalation toxicity study in rats with CNFs: need for establishing a weight-of-evidence (WoE) approach for setting no adverse exposure levels

**Figure 5.**
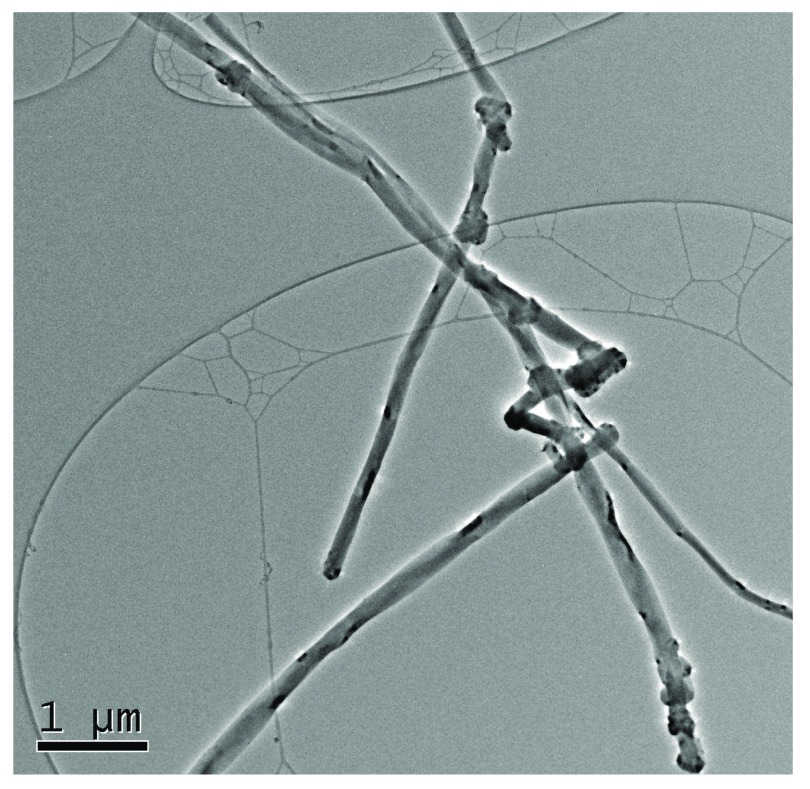
Transmission electron micrograph of an aerosolized carbon nanofiber caught on a filter.

**Figure 6.**
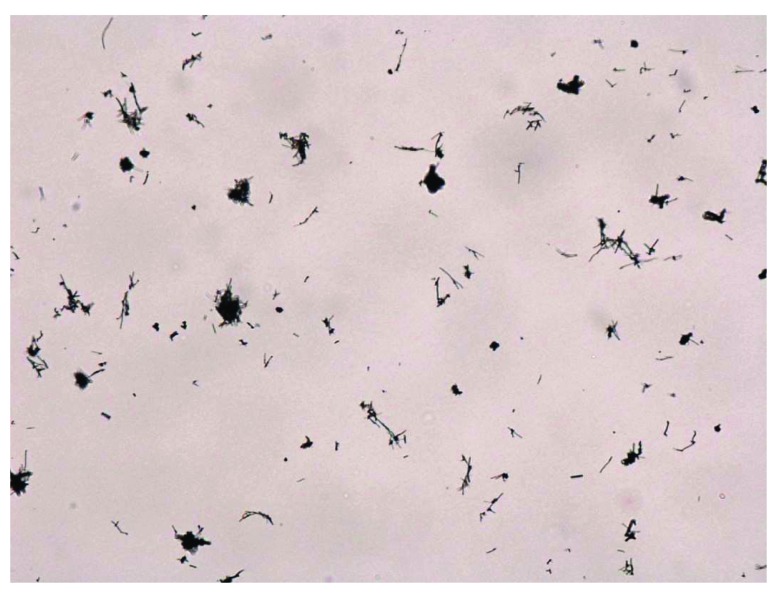
Low-magnification light micrograph of a filter used for counting aerosolized carbon nanofibers (CNFs). Note that some of the CNFs are agglomerated.

**Figure 7.**
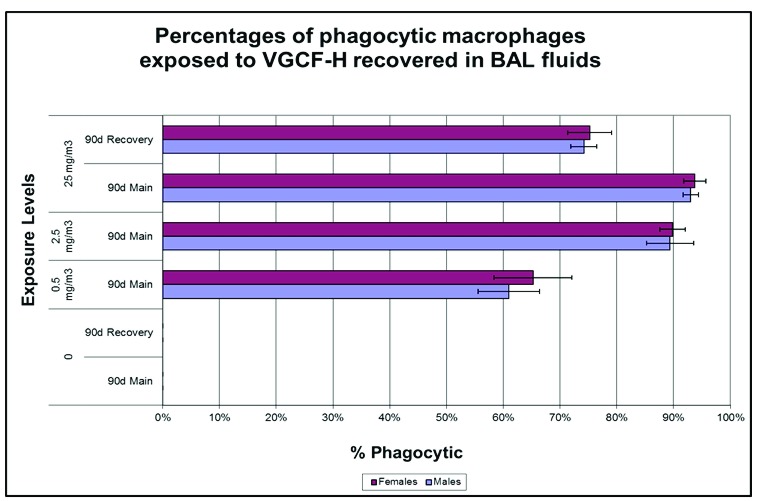
Percentages of bronchoalveolar lavage (BAL)-recovered alveolar macrophages containing carbon nanofiber (CNF) following 90 days of exposure. It is noteworthy that >90% of alveolar macrophages contained particles following 90 days of exposure to 2.5 or 25 mg/m ^3^ CNF. VGCF, vapor-grown carbon fibers.

**Figure 8.**
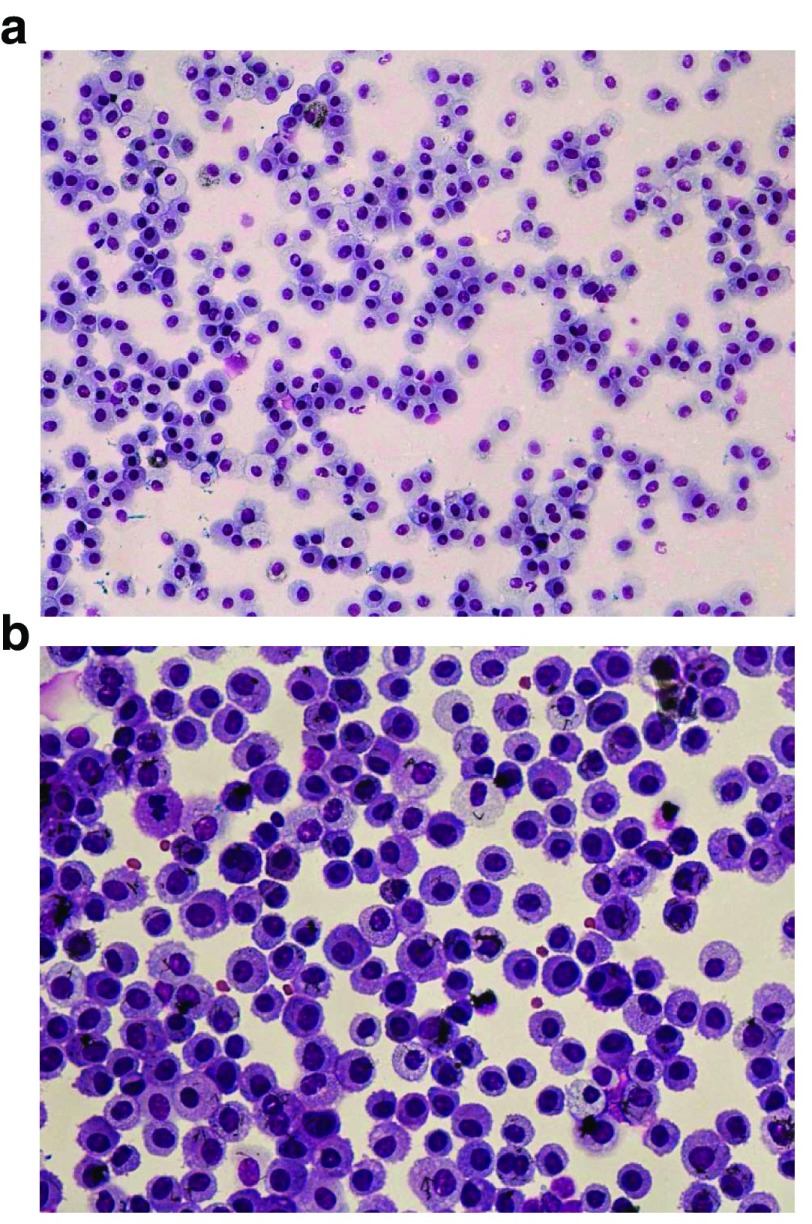
Cytocentrifuge preparations of lung cells recovered by bronchoalveolar lavage. Figure 8a represents a lower-magnification micrograph of cells recovered from an air-exposed control rat (20x magnification). Figure 8b demonstrates a high-magnification micrograph recovered from a rat exposed to 0.5 mg/m ^3^ carbon nanofiber. Note the lack of neutrophilic inflammation following 90 days of inhalation exposure.

**Figure 9.**
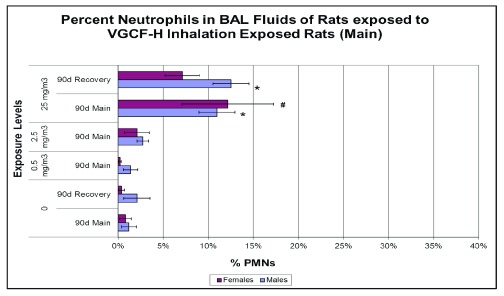
Pulmonary inflammation in carbon nanofiber-exposed rats and controls as shown by the percentages of neutrophils in bronchoalveolar lavage (BAL) fluids following 90 days of exposure. Only exposures to 25 mg/m ^3^ produced sustained pulmonary inflammatory responses of 11–12% neutrophils. * *p*<0.05. PMNs, polymorphonuclear leukocytes; VGCF, vapor-grown carbon fibers.

The goal of this subchronic study was to evaluate the long-term toxicity of inhaled VGCF™-H CNFs ( Figure 5 and Figure 6) in male and female SD rats over a 3-month period ^16^. Figure 5 demonstrates a representative transmission electron micrograph of an aerosolized CNF caught on a filter. Figure 6 represents a light micrograph of some aerosolized CNFs on a filter that were utilized for exposure quantification. Groups of male and female rats were exposed nose-only, 6 hours per day, 5 days per week to target concentrations of 0, 0.54, 2.5, or 25 mg/m ^3^ CNFs over a 90-day, 13-week exposure period. In addition, male and female rats exposed to 0 and 25 mg/m ^3^ (the highest concentration) were also evaluated at 3-months post-exposure (i.e. recovery) by clinical and histopathological methods, bronchoalveolar lavage (BAL) analysis, and epithelial cell turnover effects. Cell proliferation (CP) studies with BrdU were conducted to gauge different anatomical compartments of the respiratory tract, including the following anatomical regions of the respiratory tract: terminal bronchiole (TB), alveolar duct (AD), and subpleural regions. The results demonstrated that aerosol exposures of rats to 0.54 (4.9 f/cc), 2.5 (56 f/cc), and 25 (252 f/cc) mg/m ^3^ of VGCFTM-H produced concentration-related small, detectable accumulation of extrapulmonary fibers (outside the respiratory tract) with no adverse tissue effects outside of the lungs. Histopathological observations revealed that at the two highest concentrations tested, minimal (2.5 mg/m ^3^) and slight (25 mg/m ^3^) neutrophilic-based inflammation was observed at the anatomical junctions of the TBs and ADs (known as the AD bifurcations). These were the same anatomical sites wherein fiber-containing lung macrophages had migrated and accumulated. The impact of this exposure at high concentrations was described histologically by accumulations of neutrophilic inflammatory cells and some thickening of interstitial compartments and hypertrophy/hyperplasia of pneumocyte type II epithelial cells and was graded as slight for the rats exposed to the 25 mg/m ^3^ (highest) concentration. Lung lavage fluid and CP endpoint increases versus air-exposed controls were quantified at 25 mg/m ^3^ CNFs but were not different from control values at the 0.54 or 2.5 mg/m ^3^ exposure concentrations. It is noteworthy that greater than 90% of CNF-exposed, lavage-recovered pulmonary macrophages from rats exposed to 25 and 2.5 mg/m ^3^ CNFs had phagocytized CNFs (>60% for 0.54 mg/m ^3^). The percentages of phagocytic macrophages exposed to CNFs recovered in BAL fluids are enumerated in Figure 7. Figures 8a and 8b illustrate cytocentrifuge preparation of lung cells recovered by BAL. A non-specific nasal inflammatory response was also observed histopathologically. It was concluded that pulmonary macrophage accumulation in the pulmonary alveolar compartment likely resulted in the observation of reduced inflammatory responses in rats exposed to concentrations of 2.5 mg/m ^3^ CNFs. Alternatively, the low-level pulmonary tissue alterations at this intermediate exposure level of 2.5 mg/m ^3^ can be regarded as relatively normal physiological responses to subchronic inhalation exposures of particulates. The histopathological profile as well as the BAL fluid (biochemical results) and lung parenchymal cell turnover findings were consistently similar at the high-exposure concentration (i.e. 25 mg/m ^3^), but at the intermediate concentration (2.5 mg/m ^3^) there was a lack of compatibility when reconciling the histopathological observation of minor lung inflammation to the more sensitive BAL fluid (both inflammatory and cytotoxicity data) and cell turnover findings ( Figure 9). Therefore the investigators have suggested that a weight of evidence (WoE) approach should be implemented as the paradigm principle and criteria for describing study results or impacts and establishing no-effect levels. The implementation of these analyses and consideration of the full study database are important constructs for study assessment and corresponding determination of adverse effect levels in subchronic inhalation studies with nanoparticulate materials.

## Nanosafety research: are we on the right track?

Krug evaluated the scientific literature on nanosafety over a period of 10–15 years (during the period of 2000–2014) by undertaking an assessment of more than 10,000 publications concentrating on either human health effects or biological endpoints in both *in vivo* and *in vitro* studies (i.e. cell cultures) by focusing on four core topics ^17^. The assessment focused on three uptake pathways *in vivo*: the respiratory tract, the gastrointestinal tract, and the dermal pathways, i.e. skin. A fourth and major target was the comparability of findings following inhalation versus intratracheal instillation exposures to better gauge pulmonary exposures.

The major findings from this comprehensive evaluation of the scientific literature was that following pulmonary exposures, only a minor fraction of the inhaled or instilled dose translocated from the lung to the pulmonary or systemic circulation and ultimately reached secondary organs within the body. Moreover, the very small fraction of studies that claimed nano-specific effects related to toxicokinetic distribution could not be attributed to so-called nano effects owing to flaws in study design.

When comparing particle results of lung instillation studies versus inhalation studies (more relevant route of exposure), the findings of instillation studies were necessarily conducted at high dose overload (bolus) conditions. Krug concluded that these pulmonary inflammation effects following particle deposition were not related to a specific “nano particle” effect but occurred in response to particle exposures, per se.

A third aspect noted by Krug was that engineered nanomaterials (i.e. metals) can dissolve either slowly or more rapidly in body fluids following exposures. This has often been misinterpreted as a form of nanotoxicity but can relate to either solubility or a more general form of toxicity that is not unique to nanoparticles.

A fourth major finding by Krug was that many, if not most, of the published studies did not have adequate physicochemical characteristics of the test nanoparticle—and, in the author’s opinion, this critical shortcoming limits the impact or reliability of the findings—rendering the results “totally meaningless”.

## Biokinetics

In a series of three publications, Kreyling and coworkers studied the biokinetics and translocation or biodistribution of a single dose of nanoscale TiO _2_ particles in rats following exposures via three main routes, namely intravenous, intratracheal (pulmonary), and oral exposure (intragastric intubation) ^18– 20^. The investigators used radiolabeled TiO _2_ nanoparticles which permitted monitoring the overall biodistribution of radiolabeled TiO _2_ into various tissues over 28 days post-exposure and to implement accurate mass balance estimates. These studies represent “state-of-the-art” approaches to determine the fate and clearance of nanoparticles following various routes of exposure. It was noteworthy that the biodistribution and biokinetics of nanoscale TiO _2_ exposures following pulmonary and gastrointestinal routes were relatively similar but distinctly different from the intravenous route of administration.

## Conclusions

In conclusion, this mini-review has focused on select hazard and risk assessment strategies and research efforts that have been proposed or conducted during the past decade with the intention of better estimating potential adverse health effects following nanoparticle exposures. Although some research progress has been made on the “conceptual” and organizational front, the challenges of assessing the health effects of specific nanoparticle types remain a daunting task. Below is a listing of take-home messages that should be considered critical for future research endeavors:

- Robust and sufficient characterization of the material is an essential requirement before toxicity studies can begin
- Standardized protocols and guideline studies are necessary for the validation of methods and research results
- Round-robin inter-laboratory studies are necessary for the validation of test methods and toxicity results
- The funding for establishing protocols and standardizing methodologies should be derived from governmental organizations
- It is necessary to test commercially relevant nanoparticle types (to which humans will be exposed): hazard studies using laboratory-made or exotic-type nanoparticles have little value for determining relevant health effects
- The results of acute-type studies conducted at high doses provide little useful hazard information
- Dose-response characterization at relevant dose levels for both *in vitro* and *in vivo* studies concomitant with time-course assessments that span acute to chronic exposure durations are critically necessary to gauge the medium- and/or long-term effects of relevant nanoparticle exposures
- Given that nanoparticle-based hazard studies will inevitably be transitioned from the use of experimental animals to animal alternatives, it is necessary to develop a more effective relevant protocol to transition from *in vivo* effects to *in vitro* effects; this requires the selection of relevant cell types and cell models that reflect the route of human exposures and should be validated by a variety of research groups
- Development is required for *in vitro* toxicity methodologies or cell culture techniques to better simulate longer-term effects, including more realistic dosimetry, repeated dosing schedules, and better particle characterization techniques
- From a regulatory standpoint, more useful definitions for defining nanomaterials are necessary, as the current European Union definition is arbitrary and has little significance with respect to health effects; indeed, any definition needs to be supported by a reliable method of assessment
- The question of whether nanoparticles are more hazardous than fine particles of similar or identical chemistry has not been adequately addressed; this is important because there is a substantial database informing on the toxicity of numerous fine particle types, and this could obviate the need to conduct extensive studies on the nanoparticle forms of fine particles, for which there are significant toxicity data available
- Greater research emphasis should be focused on the biokinetics/toxicokinetics of nanoparticles at relevant human exposure levels when considering the major routes of nanoparticle exposures (i.e. pulmonary, oral, and dermal); the studies by Kreyling and coworkers have been highlighted for nano TiO _2_ exposures but need to be conducted for many other nanoscale materials
- The application of proper benchmark control particles or nanoparticles is necessary to improve the interpretation of toxicological outcomes

As discussed throughout this mini-review, a significant amount of planning and effort has been given to the challenge of establishing safety following exposures to nanomaterials and, also acknowledged, some progress has been made regarding the standardization of research protocols and generation of useful data. A number of European FP7-sponsored projects have provided some successful data-generated outcomes for hazard assessments. The NanoCare project has also been very successful in providing useful toxicological data, demonstrating that dermal exposures to nanomaterials are generally safe. Significant concerns should be raised, however, by Krug’s evaluation of more than 10,000 publications, demonstrating significant deficiencies in many toxicological publications. In taking a “snapshot” of the status of nanomaterial safety efforts, it seems clear that numerous challenges yet remain in providing accurate health and environmental decisions on a variety of nanomaterial types. Significant improvements can be made in the near term to advance better information on 1) the various different routes of exposure (pulmonary, oral, and dermal), 2) better, standardized nanomaterial characterization issues, including a better definition for nanomaterial, that can be scientifically justified, 3) improvements in research methodologies, protocols, and implementation of studies, such as time-course and dose-response issues (because acute high-dose exposure protocols generate meaningless data), and 4) the development of robust *in vitro* methodologies that can be validated for representing *in vivo* effects in standardized guideline toxicity testing.
